# Mechanisms and component design of prosthetic knees: A review from a biomechanical function perspective

**DOI:** 10.3389/fbioe.2022.950110

**Published:** 2022-09-15

**Authors:** Wei Liang, Zhihui Qian, Wei Chen, Hounan Song, Yu Cao, Guowu Wei, Lei Ren, Kunyang Wang, Luquan Ren

**Affiliations:** ^1^ Key Laboratory of Bionic Engineering, Jilin University, Ministry of Education, Changchun, China; ^2^ School of Science, Engineering and Environment, University of Salford, Salford, United Kingdom; ^3^ School of Mechanical, Aerospace and Civil Engineering, University of Manchester, Manchester, United Kingdom

**Keywords:** prosthetic knee, transfemoral prosthesis, knee mechanisms, passive knee, above-knee prosthesis

## Abstract

Prosthetic knees are state-of-the-art medical devices that use mechanical mechanisms and components to simulate the normal biological knee function for individuals with transfemoral amputation. A large variety of complicated mechanical mechanisms and components have been employed; however, they lack clear relevance to the walking biomechanics of users in the design process. This article aims to bridge this knowledge gap by providing a review of prosthetic knees from a biomechanical perspective and includes stance stability, early-stance flexion and swing resistance, which directly relate the mechanical mechanisms to the perceived walking performance, i.e., fall avoidance, shock absorption, and gait symmetry. The prescription criteria and selection of prosthetic knees depend on the interaction between the user and prosthesis, which includes five functional levels from K0 to K4. Misunderstood functions and the improper adjustment of knee prostheses may lead to reduced stability, restricted stance flexion, and unnatural gait for users. Our review identifies current commercial and recent studied prosthetic knees to provide a new paradigm for prosthetic knee analysis and facilitates the standardization and optimization of prosthetic knee design. This may also enable the design of functional mechanisms and components tailored to regaining lost functions of a specific person, hence providing individualized product design.

## 1 Introduction

The prescription criteria and selection of prosthetic knee depend on the interactions between the patient and prosthesis. The function levels of people with above-knee amputations can be categorized into five levels (Table), according to the capacity of the patient to perform daily life tasks independently (Burnfield et al., 2012). During level walking, the fundamental function of a knee prosthesis is to support the body weight and dissipate energy. On this basis, prosthetic knees, including passive, microprocessor-controlled, and powered knees, have been compared and summarized, from the aspects of design, performance assessment, and control strategies (Michael, 1999; Torrealba et al., 2008; Grimmer and Seyfarth, 2014; Price et al., 2019).

Passive knees, or the so-called mechanical knees, mainly rely on mechanical structures. Review articles about passive knees concentrate on polycentric mechanisms and structure optimization. The design, modeling, kinematics, and stability of a knee that was based on a four-bar linkage have been a main topic of prosthesis research for the past decade (Andrysek, 2010; Amador et al., 2011; Anand and Sujatha, 2017; Andrysek et al., 2019; Mohanty et al., 2020; Soriano et al., 2020). Microprocessor-controlled knees (MPKs) can automatically adjust damping characteristics through external sensors and servo valves, which provide a wider range of self-selected speeds and augmented stability. The reviews of MPKs focus on state-of-the-art devices that regulate stance and swing phase resistance, which have been illustrated in terms of electronic sensors and complex control algorithms (Martin et al., 2010; Thiele et al., 2014; Fluit et al., 2020). Furthermore, powered knee prostheses, or so-called active prosthetic knees (APKs), are assembled with actuators to inject energy at the knee joint and can be used on different terrains, including rough roads, stairs, and ramps. In recent review articles, the APK of actuator mechanisms, actuation principles, control strategies, efficiency assessments, performance metrics, and their limitations have been studied (Laferrier and Gailey, 2010; Tucker et al., 2015; Windrich et al., 2016; Pieringer et al., 2017; Lara-Barrios et al., 2018; Torrealba and Fonseca-Rojas, 2019).

From these reviews, it is known that a prosthetic knee is composed of two main parts: 1) functional components: the elements for energy reservation (spring), dissipation (damper), and generation (motor); and 2) functional mechanisms: the mechanical frame for stability, motion, and adjustment. On the one hand, functional components can determine the characteristics of the knee. In semiactive knees, for example, electrical motors usually work with springs (Martinez-Villalpando and Herr, 2009; Flynn et al., 2015), hydraulic actuation systems (Lambrecht and Kazerooni, 2009; Lee et al., 2020), or magnetorheological dampers (Park et al., 2016) to adapt to various terrains. However, functional mechanisms can change the functions of the knee. For instance, the five-bar redundant mechanism (Awad et al., 2012; Lenzi et al., 2018), or motorized clutch (Rouse et al., 2014) Lenzi et al., 2015) in semiactive knees, switches the operating mode or modulates the joint impedance of the prosthetic knee. From the aspect of functional components and mechanisms, individuals with transfemoral amputation can choose suitable knees, and prosthesis engineers can design efficient mechanisms. However, a few of recent reviews have analyzed the effects of passive components and structures, while the basic principles of prosthetic knees are still indistinct to users, prosthetists, and developers.

Typically, a gait cycle has a stance phase and swing phase, which can be further divided into five subphases, including stance flexion (early stance), stance extension (middle stance), preswing (late stance), swing flexion (early swing), and swing extension (late swing) (Rose and Gamble, 2006). The stability and resistance of a prosthetic knee are key functions required for a safe yet natural walking gait. The desired motion and torque of a prosthetic knee in different phases are intrinsically associated with their functional structures and components. Users of different activity levels will choose the knees with different functional structures and components (Table 1). Based on this point, we attempt to explain the basic functions of mechanical knees with the following related walking requirements: stance stability, stance flexion, and swing resistance.

**TABLE 1:** Transfemoral amputation functional classification levels.

K-level	Descriptor	Knee	Required functions
**K0**	This patient does not have the ability or potential to ambulate or transfer safely with or without assistance, and a prosthesis does not enhance his or her quality of life or mobility.	Not eligible for prosthesis	—
**K1**	This patient has the ability or potential to use a prosthesis for transfers or ambulation on level surfaces at fixed cadence—a typical limited or unlimited household ambulator.	Single-axis, constant-friction knee	Walking stability
**K2**	This patient has the ability or potential for ambulation with the ability to traverse low-level environmental barriers, such as curbs, stairs, or uneven surfaces—a typical community ambulator.	Single-axis, constant-friction knee	Walking stability and swing resistance
**K3**	This patient has the ability or potential for ambulation with variable cadence—a typical community ambulator with the ability to traverse most environmental barriers and may have vocational, therapeutic, or exercise activity that demands prosthetic use beyond simple locomotion.	Fluid and pneumatic-control knees	Walking stability, swing resistance, and early stance flexion
**K4**	This patient has the ability or potential for prosthetic ambulation that exceeds basic ambulation skills, exhibiting high impact, stress, or energy levels—typical of the prosthetic demands of the child, active adult, or athlete.	Any are appropriate	Walking stability, swing resistance, and early stance flexion

This review aims to 1) bridge the knowledge gap between the basic demands of individuals with above-knee amputation and the functional structures of knee devices; 2) guide developers by highlighting general features and performance criteria of prosthetic knees; and 3) optimize the design of prosthetic knees with lightweight and compact structures.

This article is organized as follows: in Section 2, the methods in the literature review are introduced; in Section 3, the biomechanical challenges during walking are introduced; in Section 4, we discuss the stability mechanisms of the knee joint; in Section 5, we describe the cushion structures in a prosthetic knee based on early-stance-flexion (ESF) motion; in Section 6, the functional components acting on the knee axis for modulating swing movement are analyzed; in Section 7, the general trends based on the functional mechanisms for the remaining problems of a prosthetic knee are discussed; and conclusions are provided in Section 8 of the article.

## 2 Methods

### 2.1 Search strategy

A literature search was conducted until 1 June 2022 in eight English databases following PRISMA method. The used databases are Web of Science, Springer, Wiley, Science direct, IEEExplore, ASME, PubMed and Google Scholar. In addition, patents of passive prosthetic knee were explored *via* Google Patents. Eight English keywords, including “above-knee prosthesis,” “transfemoral knee prosthesis,” “prosthetic knee mechanism,” “passive prosthetic knee,” “brake prosthetic knee,” “polycentric prosthetic knee,” “mechanical knee,” or “transfemoral amputation,” are used during database retrieves. The beginning date and end date of these database searches were set from January 1, 1950 to the latest date provided by the databases.

Furthermore, a manual search was performed on three types of publications from the screened results of the database searches. The first type of publication was review articles, the second type was research articles of functional structure in mechanical knees, and the other type of publication was clinical studies of transfemoral amputation. Finally, 140 results of the manual searches were screened, including 113 journal articles and 27 patents.

### 2.2 Exclusion criteria

**FIGURE 1:**
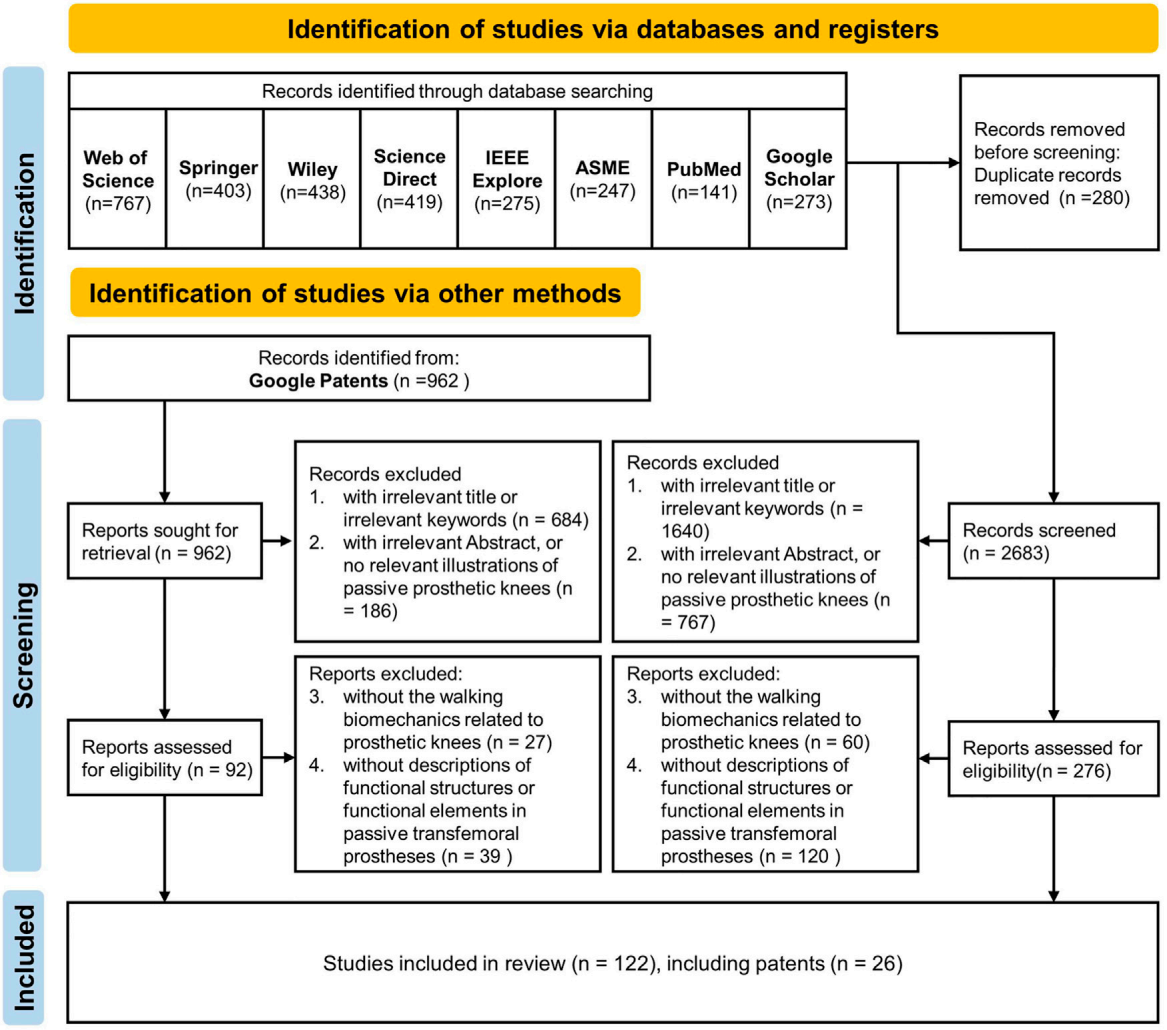
Flowchart of database search and manual search based on PRISMA.

Any records that met the following four levels of criteria were deleted: 1) with irrelevant title or irrelevant keywords; 2) with irrelevant abstract or no relevant illustrations of passive prosthetic knees; 3) without the walking biomechanics related to prosthetic knees; and 4) without descriptions of functional structures or functional elements in passive transfemoral prostheses. The database and manual search and screening procedures are illustrated in the flowchart in Figure 1.

### 2.3 Classification criteria

Passive knee prostheses from the screened publications and online information were classified based on the biomechanical challenges of persons with transfemoral amputation, namely, falls, osteoarthritis, and gait asymmetry.

A fall is mainly related to stance stability, which is the basic requirement of safety for all individuals in the K0–K4 levels. Stance stability is realized by functional structures such as four-bar linkages or by functional components, such as hydraulic units.

Osteoarthritis corresponds to stance flexion, which is desired by active users in the K3 and K4 levels. Stance flexion can reduce the impact from the ground and improve the comfortability of the residual limb. It depends on the functional structures of the prosthetic knee and allows for a limited flexion angle at the early-stance phase without losing stability.

Gait asymmetry is associated with swing resistance, where this essential function controls the maximum flexion angle and determines the timing of full extension. Swing resistance is regulated by the functional components that act on knee axis.

**FIGURE 2:**
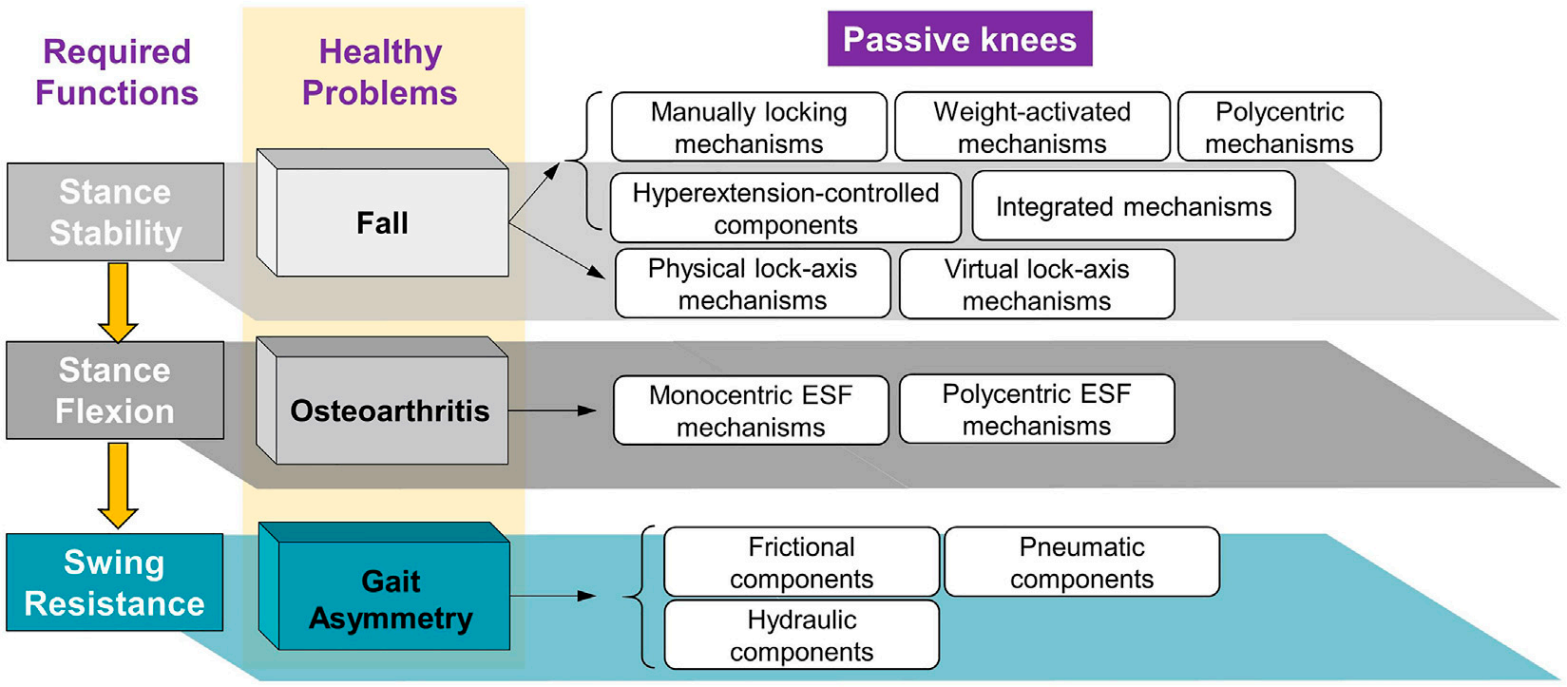
Framework based on the required functions of prosthetic knee on the aspect of required functions during level walking.

Mechanisms and components in knee prostheses are closely related to basic walking functions. Therefore, the biomechanical challenges and required functions of the knee joint are proposed first (Figure 2). Then, as the key solutions to those health problems, the functional structures and components of current passive prosthetic knees are illustrated. We wish to provide a better understanding of the basic functional principles of knee prostheses based on this framework.

## 3 Biomechanical challenges

Problems resulting from above-knee amputation are related to the biomechanics of human walking. This section discusses the causes of these problems and interactions with prosthetic knees.

### 3.1 Fall and stability

Stance stability is the prevention of falls at the early-stance phase and allows for flexion at the preswing phase. Above-knee amputation is intrinsically associated with an increased risk of falling (Miller et al., 2001). Even with knee prosthetic intervention, the probability of falling for an individual with amputation is 82% per year, in which the average number of falls per person per year is 3.9 (Kahle et al., 2008; Wong et al., 2015). According to statistics, the incidence risk factors for falling vary greatly among different kinds of prosthetic knee users (Liu et al., 2017). This suggests that the decreased stability is closely related to the knee joint, and the prosthetic knee is a major determinant of falling. Two reasons for falling caused by prosthetic knee joints were introduced (Hisano et al., 2020), i.e., buckling in the stance-flexion phase and stumbling in the preswing phase. In both cases, prosthetic knee stability is affected by the position and direction of the load line.

**FIGURE 3:**
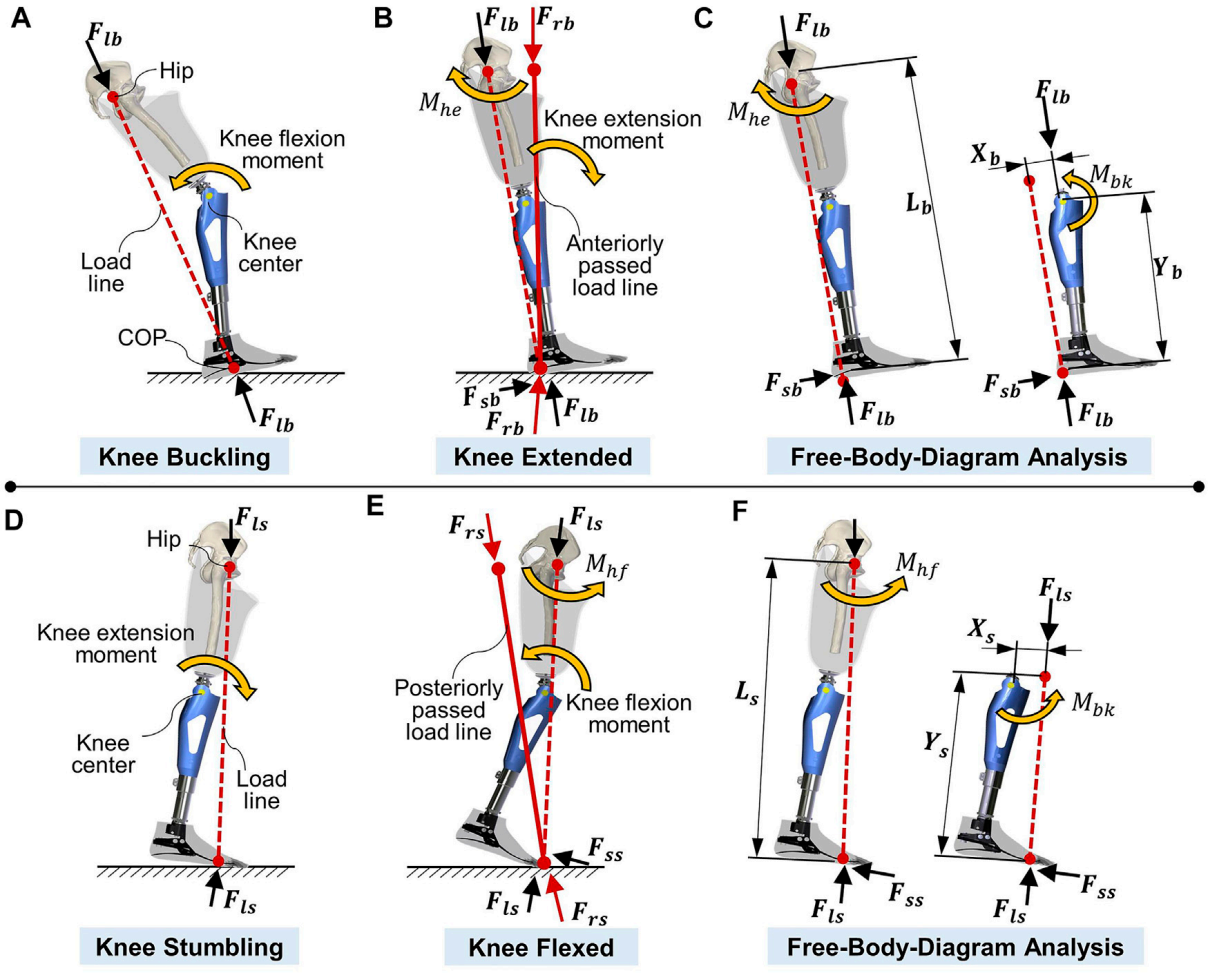
Prosthetic knee at early-stance and late-stance. **(A)** Knee buckling with load line (dash line) passes posteriorly to the knee center at early-stance. **(B)** Knee stabilizes by the hip extension moment with load line transfers anteriorly (solid line) to knee center. **(C)** Free body diagram (FBD) analysis of the minimum hip moment required for the knee stabilization. **(D)** Knee stumbling with load line (dash line) passes anteriorly to the knee center at late-stance. **(E)** Knee stabilizes by the hip flexion moment with load line transfers posteriorly (solid line) to knee center. **(F)** FBD analysis of the minimum hip moment required for the knee flexion.

Figures 3A–C demonstrate the falling risk at heel strike. Without the hip extension moment, the load force acts on the hip joint center and the center of pressure (COP) of the foot to form a load line (the red dashed line). The posteriorly located load line causes a flexion moment at the knee and buckling motion at the early-stance phase. In contrast, if an extension moment is exerted by the hip joint, a shear force will be applied on the foot. The resultant force shifts the load line forward (the red solid line), which exerts an extension moment and stabilizes the knee joint. The free-body diagram (FBD) method is then used to calculate the minimum value of the required hip extension moment for stabilizing the knee joint from the view of the whole residual side:

$$M_{he}=\frac{L_{b}}{Y_{b}}\left(F_{lb}X_{b}-M_{bk}\right)$$ (1)

Figures 3D–F demonstrate the stumbling risk during the toe-off phase. A prosthetic leg can swing only if the load line passes posteriorly to the knee center. The load line must be shifted backward (from the red dashed line to the red solid line) with a hip flexion moment, exerting a flexion moment on the knee, which is thus able to flex. Similarly, the flexion moment of the residual hip joint determines how easy it is to flex the prosthetic knee, and the FBD method is used to calculate the minimum required flexion hip moment as

$$M_{hf}=\frac{L_{s}}{Y_{s}}\left(F_{ls}X_{s}+M_{bk}\right)$$ (2)

**FIGURE 4:**
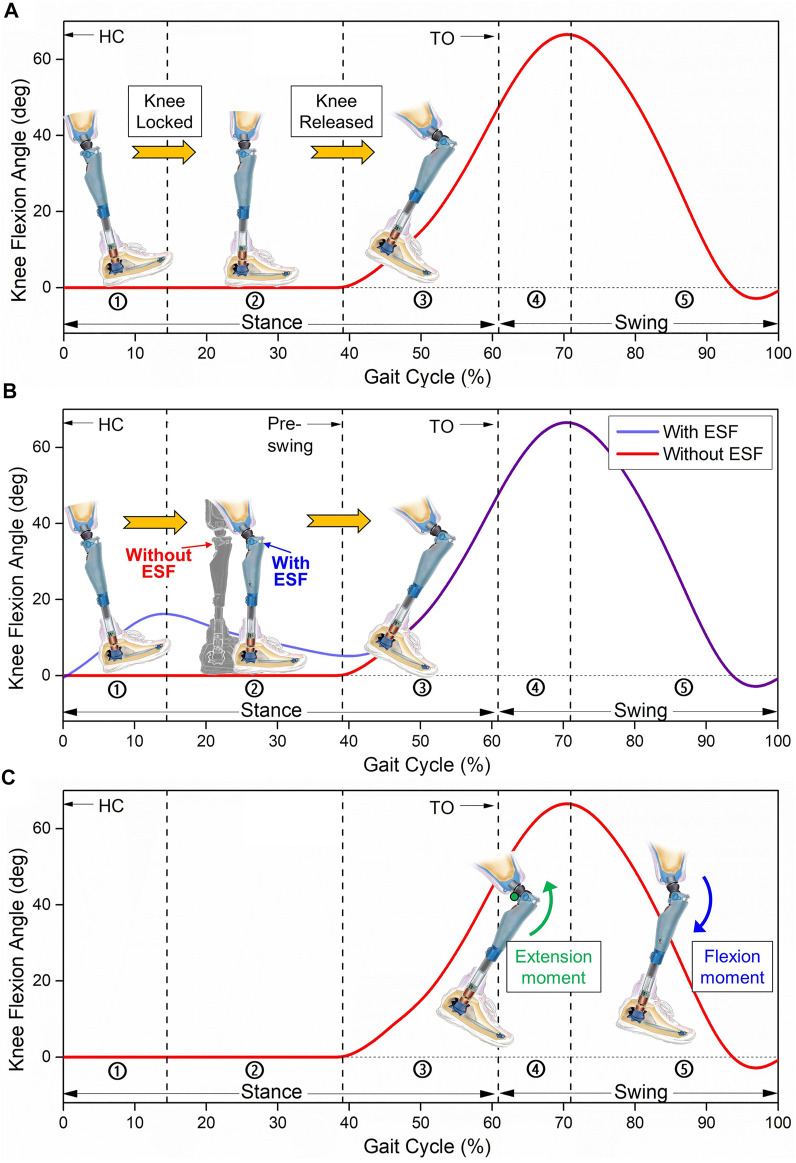
Function requirements of passive knees. **(A)** Diagram of target knee flexion angle and states of monocentric knee during stance phase, in which the phases from ① to ⑤ represent early stance, middle stance, preswing, early swing, and late swing, respectively. **(B)** Comparison of prosthetic knees with and without ESF during stance phase. **(C)** Knee moment required for a smooth yet natural swing phase.

Because the load force (*F*_*lb*_ or *F*_*ls*_) and leg length (*L*_*b*_ or *L*_*s*_) are dependent on an individual’s physical condition, three factors in prosthetic knee are considered as variants, including the knee brake moment (*M*_*bk*_), the distance between the load line and the joint center (*X*_*b*_ or *X*_*s*_), and the vertical height of the knee joint center (*Y*_*b*_ or *Y*_*s*_). To prevent falls during the stance phase, the braking moment and the position of the knee center must be properly designed. The features of stability of the prosthetic knee are all governed by the knee-axis-based functional mechanisms and components (Figure 4A).

### 3.2 Osteoarthritis and early-stance flexion

In addition to stability, the impact absorption capacity is important when evaluating a prosthetic knee. ESF can effectively reduce the impact exerted at heel strike for the K3- and K4-level amputees. It can protect the human joints of active users from fatigue and damage.

It is well accepted that osteoarthritis is associated with the long-term use of prosthetic limbs (Gailey et al., 2008). Nearly 63% of users have osteoarthritis in their residual limb (Burke et al., 1978; Mussman et al., 1983; Kulkarni et al., 1998). This health problem results from the motion differences between the intact limb and residual limb. After the toe-off phase, the leg is required to swing forward and lift upward, where the inertia of the leg will convert into kinetic energy at the next strike. Accordingly, the leg has to brake the movement and attenuate the impact for support. In fact, the breaking impulse of the prosthetic side is much lower than that of the intact side (Houdijk et al., 2009). The main reason is that the extensor muscles of healthy knee joints contribute to shock absorption and leg braking with a flexion angle, which is known as the ESF of knee (Figure 4B) (Nolan and Lees, 2000; Beyaert et al., 2008). However, prosthetic knees are normally locked during the stance phase for stability, and ESF is not allowed. The loss of function is compensated by the intact side, which has 20% longer supporting time and absorbs twice the impact (Gard, 2006; Hof et al., 2007; Castro et al., 2014). Consequently, forces and impulses are repetitively applied to the intact limb, leading to greater incidences of osteoarthritis (Kulkarni et al., 1998).

### 3.3 Energy expenditure and gait symmetry

Compared to healthy knees, prosthetic knees cannot swing naturally without motion regulating components. The knee exerts torque to affect its kinematics, which mainly generates negative work. To improve the efficiency and naturality of prosthetic knees, frictional, pneumatic, and hydraulic units are proposed to work on the knee-axis.

Individuals with transfemoral amputation have a less efficient gait and 27–88% increase in metabolic cost (Gitter et al., 1995; Hoffman et al., 1997). High energy expenditure is mainly caused by the asymmetric gait from prosthesis motion and loading (Jaegers et al., 1995). The symmetry index of the joint angle and torque of an intact leg and prosthetic leg have been compared, where the value of the prosthetic knee was lower than that of the hip or ankle (Crenshaw and Richards, 2006; Kaufman et al., 2012). Great torque is required for the residual hip to stabilize the knee during stance (Czerniecki, 1996; Highsmith et al., 2011), while there is a strong need for proper energy flow during swing, including the extension moment to prevent excessive heel rise and the flexion moment to prevent shank acceleration (Figure 4C) (Inman, 1967; Dillingham et al., 1992).

## 4 Mechanisms for stance stability

### 4.1 Monocentric knee

Typically, monocentric knees use functional structures to control the parameter *M*_*bk*_ in Eqs 1, 2, thus ensuring safety at the stance phase. The brake moment should be able to vary automatically to accommodate knee motion during the stance phase of the gait cycle (Figure 4A). The required torque of the residual hip is thus minimized, while stability during stance is guaranteed. The brake moment can be activated/deactivated by manual operation, by body-weight load, or by knee hyperextension.

#### 4.1.1 Manually locking knee

**FIGURE 5:**
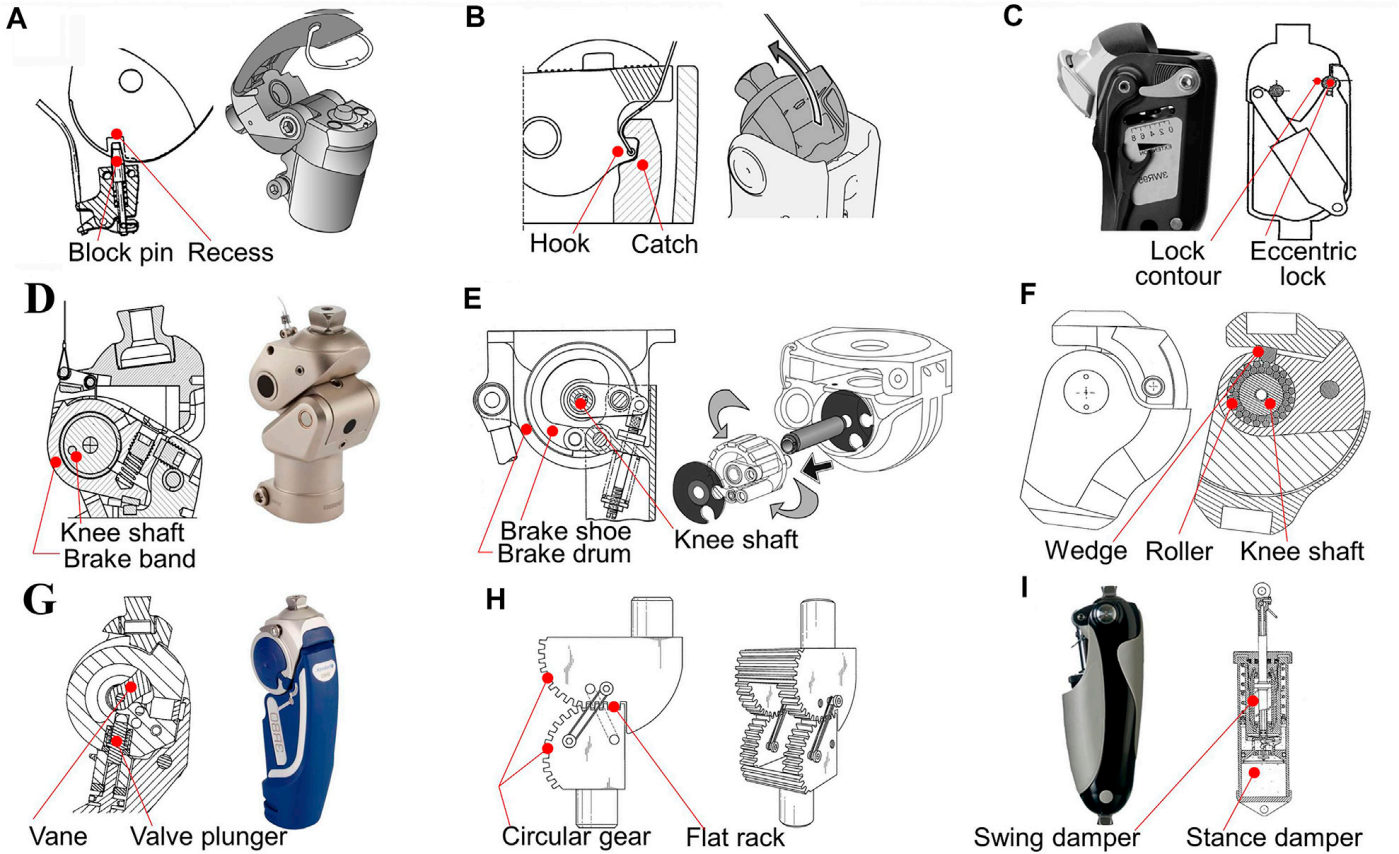
Monocentric knees for stance stability. **(A)** Manually locking knee with the block pin and recess (Blatchford^®^ Compact SAKL, Boiten et al., 2015), reproduced with permission from copyright 2017 by Blatchford^®^. **(B)** Manually locking knee with the hook and catch (Ottobock^®^ Prosedo 3R31, Haupt, 1987), reproduced with permission from copyright 1987 by Ottobock^®^. **(C)** Manually locking knee with the eccentric mechanism (Ottobock^®^ Aqua 3WR95, Bröckl and Dietl, 2015), reproduced with permission from copyright 2015 by Ottobock^®^. **(D)** Weight-activated knee with the brake shaft and band (Ossur^®^ Balance OFM2, Karlsson et al., 2015), reproduced with permission from copyright 2017 by Ossur^®^. **(E)** Weight-activated knee with the brake drum (Blatchford^®^ ESK^+^, (Blatchford and Tucker, 1980), reproduced with permission from copyright 1980 by Blatchford^®^. **(F)** Weight-activated knee with the frictional bushing (Wagner and Krukenberg, 2000), reproduced with permission from copyright 2000 by Ottobock^®^. **(G)** Weight-activated knee with the hyraulic brake (Ottobock^®^ 3R80, Wagner and Krukenberg, 1998), reproduced with permission from copyright 1998 by Ottobock^®^. **(H)** Weight-activated knee with the gear meshing mechanism (Ramakrishnan and Reed, 2020), reproduced with permission from copyright 2020 by South Florida University. **(I)** Hyperextension-controlled knee (Ossur^®^ Mauch, Mauch, 1968), reproduced with permission from copyright 1968 by Ossur^®^.

A manually locking knee is locked during the whole gait cycle. When the knee is locked, the value of braking moment is infinite, which minimizes the required hip extension moment at the early stance. The manually locked mechanism can only be released if a load is not applied. Typically, a release component—a pulley—is mounted on the stump socket and is used for supporting and pulling the releasing cable. The lock mechanisms in the prosthetic knee can be released by tightening the cable manually to allow knee flexion. The frequently used lock includes a pin-recess mechanism (Figure 5A) (Boiten et al., 2015), a catch-recess mechanism (Figure 5B) (Haupt, 1987), or an eccentric lock and asymmetric contour mechanism (Figure 5C) (Bröckl and Dietl, 2015). In addition, these lock mechanisms can automatically be engaged by a spring, when the joint returns to the full extension position.

A manually locking knee is particularly suitable for new or less-active users, who need the highest safety benefit. Despite the minimized hip extension moment at the early stance, the infinite value of the braking moment results in a great value of the hip flexion moment. This means that the braking moment does not disappear automatically in the preswing phase, and the prosthetic knee joint is unable to flex in the whole walking gait cycle. The user, thus, walks with a stiff-legged gait, whose hip joint must be raised to create clearance between the foot and ground. This prosthesis has been deemed an unacceptable long-term solution.

#### 4.1.2 Weight-activated knee

A smooth transition from the stance to swing phase can be achieved only if knee flexion is enabled at the late-stance stage. In the above-knee amputation gait cycle, a large knee braking moment value is required to maintain stability at an early stance, while a small brake moment is helpful to allow knee flexion at preswing. To fulfill these requirements, the weight-activated knee mechanisms are proposed.

Typically, a weight-activated knee utilizes a controlling axis to control the brake components around the knee axis. A frictional brake is most widely used in weight-activated knees, which exerts the brake moment via a frictional band and brake drum (Shorter and Aulie, 2001; Jensen and Raab, 2003). As the body weight is applied, the relative rotation of the two ends of the brake band occurs. The brake band tends to rotate and squeeze the brake drum with a braking torque. If the weight is removed, a spring is usually utilized to push the brake band into the disengage state, allowing the lower leg to rotate about the knee axis. The functional structures of the frictional brake can act in the form of a brake band with an inner brake drum (Figure 5D) (Karlsson et al., 2015), a hollow drum with an inner brake shoe (Figure 5E) (Blatchford and Tucker, 1980), and frictional bushing with rollers (Figure 5F) (Wagner and Krukenberg, 2000).

For a greater braking moment during stance, a weight-activated mechanism can be achieved by the hydraulic circuit (Figure 5G) (Wagner and Krukenberg, 1998). For personalized customization and ease of manufacturing, 3D-printed gears can be used in prosthetic knees, where the ending flat racks can be locked when weight is applied (Figure 5H) (Ramakrishnan et al., 2016; Ramakrishnan et al., 2017; Ramakrishnan and Reed, 2020).

According to the load on the residual side, weight-activated knees change the brake moment automatically, therefore enabling the joint to be locked or released at the stages in Figure 4A. However, the braking components must be carefully adjusted so that the knee can be released to flex at the right stage. In fact, a weight-activated knee can only rotate freely after part of the user’s weight is transferred to the contralateral leg. The unlocking quantity of the transferred mass is governed by the preload of the spring in the brake mechanism, which must be adjusted correctly according to the variation in the ground reaction force (GRF) during stance. Otherwise, there will still be the risk of buckling or stumbling.

#### 4.1.3 Hyperextension-controlled knee

Hyperextension control eliminates the drawback of brake moment dependency. Hyperextension control is achieved by the swing and stance (SNS) hydraulic unit, which acts on the knee axis (Figure 5I) (Mauch, 1968). In the stance damper, the hydraulic circuit is blocked by a pendulum valve, locking the knee joint normally. Unless there is a knee hyperextension motion, the valve can be opened to release the SNS unit, allowing subsequent knee flexion. It should be noted that the SNS unit will lock the knee again during flexion, if the motion is stopped.

### 4.2 Polycentric knees

**FIGURE 6:**
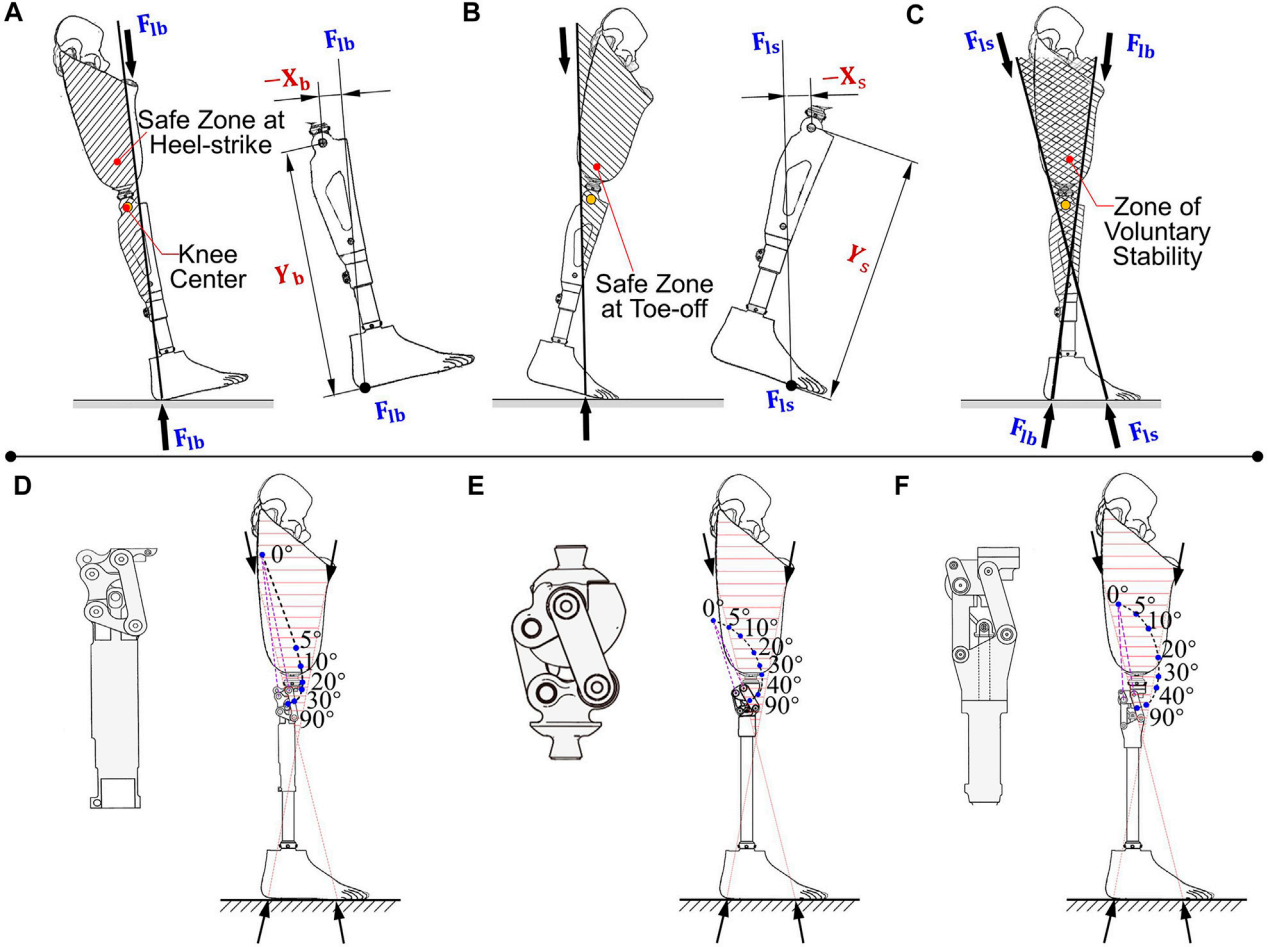
Polycentric knees for stance stability. **(A)** The safe zone at heel-strike based on load line *F*_*lb*_ *F*_*lb*_. **(B)** The safe zone at toe-off based on load line *F*_*ls*_ *F*_*ls*_. **(C)** The zone of voluntary stability, in which the knee can maintain stability at early-stance and ease knee flexion at preswing, simultaneously. **(D)** Polycentric knee with elevated instant center (Proteval^®^ Acphapend). **(E)** Hyper-stabilized polycentric knee (Ottobock^®^ 3R36). **(F)** Voluntary-controlled polycentric knee (Hosmer^®^ Spectrum).

Unlike a monocentric knee with a fixed knee center, a polycentric knee can vary the instantaneous center of rotation (ICR) during flexion. According to Eqs 1, 2, the value of *X*_*b*_/*Y*_*b*_ or *X*_*s*_/*Y*_*s*_ determines how the knee center influences the user’s minimum required effort. To quantitatively evaluate the effect during the stance phase, the concept of the “zone of voluntary stability” is introduced, which provides the ability of a prosthetic knee to simultaneously maintain stability and avoid stumble (Radcliffe, 1977; Radcliffe and Deg, 2003). The residual hip moment determines the direction of the resultant load line, which affects the stability zone at the heel-strike (Figure 6A) and at toe-off stages (Figure 6B). The zone of voluntary stability for an above-knee user is the overlapping area (Figure 6C). Then, the required hip moment can be calculated without the braking moment at stance flexion and preswing, respectively,

$$M_{he}=\frac{X_{b}}{Y_{b}}F_{lb}L_{b}$$ (3)

$$M_{hf}=\frac{X_{s}}{Y_{s}}F_{ls}L_{s}$$ (4)

Most positions of the ICR located in the zone of voluntary stability can be achieved by a properly designed four-bar linkage. The centrode, indicating the trajectory of the ICR, gives a beneficial value of *X*/*Y* at different flexion stages, therefore minimizing the required hip torque during walking. According to the centrode, polycentric knees can be categorized into three types, including elevated-instant-center, hyperstabilized, and voluntary-controlled knees (Radcliffe, 1994).

#### 4.2.4 Elevated-instant-center knee

An elevated-instant-center knee has a long anterior link and a short posterior link (Figure 6D) (Radcliffe and Deg, 2003). The elevated position of the initial knee center is conducive to reducing the required hip moment during both early-stance and pre-swing phases and is suitable for geriatric or less-active users. However, it is necessary to provide a reasonable cosmesis at a flexion of 90°, and the elevated ICR must move downward rapidly with knee flexion. The ICR will descend greatly within a flexion angle of 5°, and stability can be lost if the knee is not fully extended at heel strike.

#### 4.2.2 Hyperstabilized knee

With small changes in the length condition of the elevated-instant-center knee, dramatically different kinematic behaviors can be achieved. The initial ICR of this polycentric knee is located posterior to the zone of voluntary stability, which is known as hyperstabilized knee (Figure 6E) (Radcliffe and Deg, 2003). No hip extension moment is required to maintain early-stance stability; meanwhile, the knee cannot flex at the preswing phase, even with the maximum exerted hip flexion moment; hence, it acts the same as a manually locking knee. This characteristic makes the hyperstabilized knee a primary choice for users who ask for a high level of stability.

#### 4.2.3 Voluntary-controlled knee

The ICR of a voluntary-controlled knee smoothly moves with increasing flexion angles (Figure 6F) (Radcliffe and Deg, 2003). The centrode stays at a relatively elevated position within the stability zone, during the first 10 degrees of knee flexion. It offers the mechanical advantage for users to rotate the knee voluntarily and resist abrupt flexion. The voluntary control is beneficial for controlling stability when walking on rough ground and sloping surfaces, as well as when taking short steps. In fact, the actual ability to control motion and stability depends upon the physical capabilities of users. A voluntary-controlled knee is advantageous for active users with a desire for vigorous gaits, while it may not be optimal for less-active users, whose stability and safety are the main requirements.

Although three kinds of configurations of polycentric knees have been proposed to improve stance stability, falling still occasionally occurs in the following two situations due to insufficient residual hip moment (Andrysek et al., 2005). First, a polycentric knee does not swing to a fully extended position prior to heel strike, causing the load line posterior to its ICR. Second, the residual hip offers an insufficient extension moment, and the prosthetic knee thus cannot maintain an extended state at midstance.

#### 4.2.4 Integrated knee-axis structures

To realize higher walking capacity for less-active users, there are prosthetic knees that integrate braking action and polycentric features. For example, the DAW^®^ Sure-Stance knee consists of a four-bar linkage and frictional weight-activated brake mechanism, in which the posterior upper pivot shafts act as the brake drum surrounded by a brake clamp (Chen and Chen, 2010). Brake action can also be achieved in polycentric knees through the hyperextension-controlled units, such as in the Blatchford^®^ KX06 knee (Tang et al., 2015). With the aid of an SNS cylinder, the knee can only flex with hyperextension torque at preswing. The centrode can thus be optimized in the zone of voluntary control, which helps individuals with amputation achieve higher activity levels.

### 4.3 Ground-reaction-force affected knees

The knees in this section utilize GRF to switch the state of the knee axis, including locking and releasing. This kind of mechanism (lock axis) works together with knee-axis structures to maintain joint stability, thus further reducing the risk of falling during stance.

#### 4.3.1 Instability diagram evaluation

**FIGURE 7:**
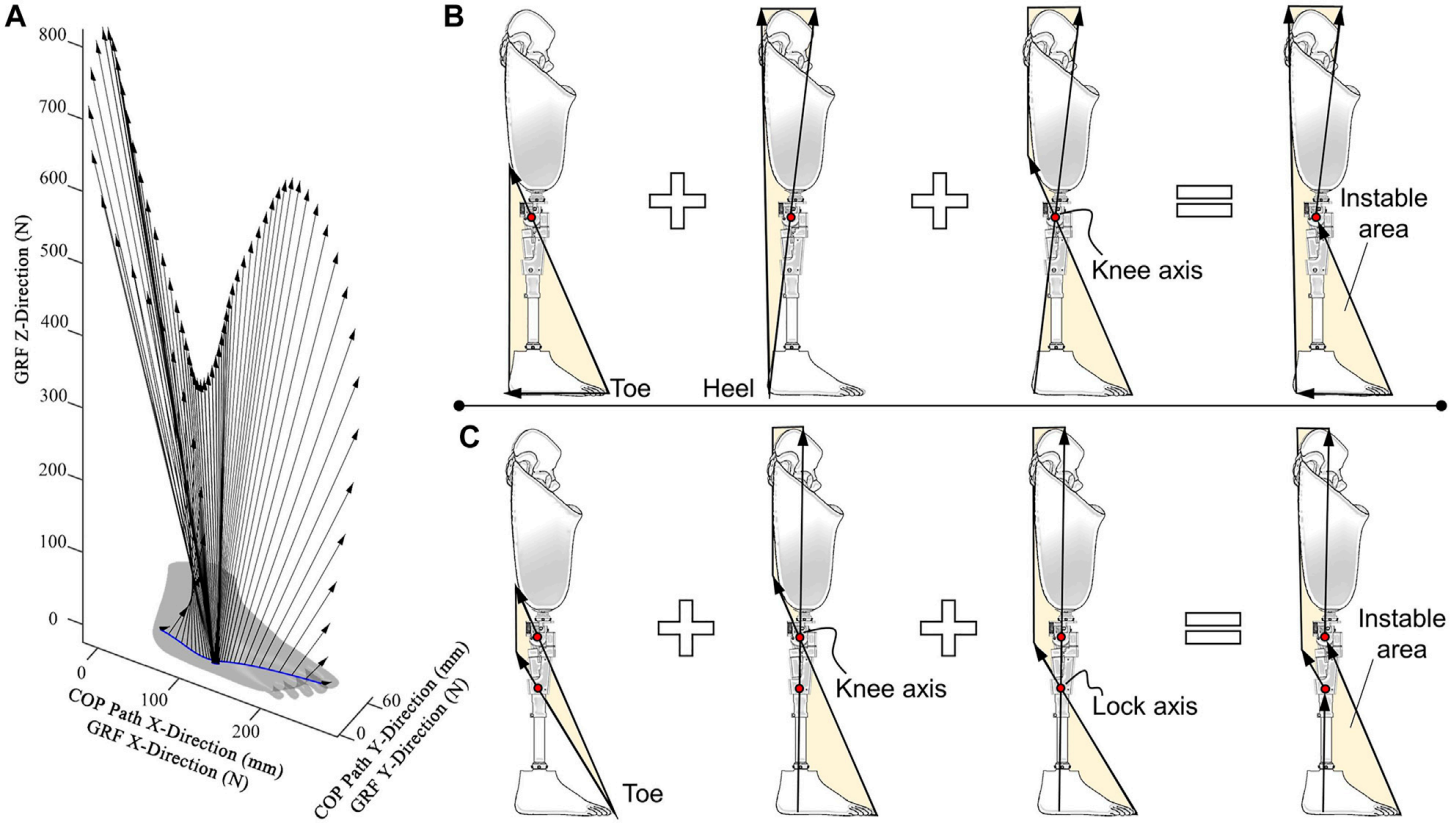
Characteristics of the knee-axis and lock-axis mechanisms. **(A)** Ground reaction force (GRF) vectors from heel-strike to toe-off during a natural gait. **(B)** Instability diagram (the yellow area) of the prosthetic knee only with the knee-axis mechanism. **(C)** Instability diagram (the yellow area) of the prosthetic knee with both knee-axis and lock-axis mechanisms.

In a natural walking gait, GRF variation corresponds to leg kinematics (Figure 7A). During stance, the origin, magnitude, and orientation of GRF vectors vary with gait phases. In particular, the COP of the foot moves from heel to toe and is associated with the engagement of the lock-axis mechanism.

According to the characteristics of GRF, the instability diagram is an effective method to illustrate the stability of the knee axis and lock axis. This method has already been used to analyze the stability characteristics of several commercially available knees (Andrysek et al., 2005). It can provide an instability zone, and when load lines are located in this zone, it will lead to knee buckling. A smaller instability zone means a higher level of safety at stance.

Based on this approach, a prosthetic knee with only knee axis is compared to that with both knee axis and lock axis. On the one hand, the instability zones of the knee axis only depend on the position of the knee center at stance (Öberg, 1983; Radcliffe, 1994). The total area of the instability zone (Figure 7B) is the sum of 1) the zone of the load lines originating at the toe that creates a flexion moment at the knee axis; 2) the zone of the load lines originating at the heel that creates a flexion moment at the knee axis; and 3) the zone of the load lines passing through the knee axis and between the toe and heel-load boundaries. On the other hand, a prosthetic knee with two stability-affecting axes can only flex if the load line passes the instability zone located between these two axes. The rear-foot loading condition is excluded from the instability zone, therefore providing a smaller instability zone (Figure 7C).

#### 4.3.2 Physical lock-axis mechanisms

**FIGURE 8:**
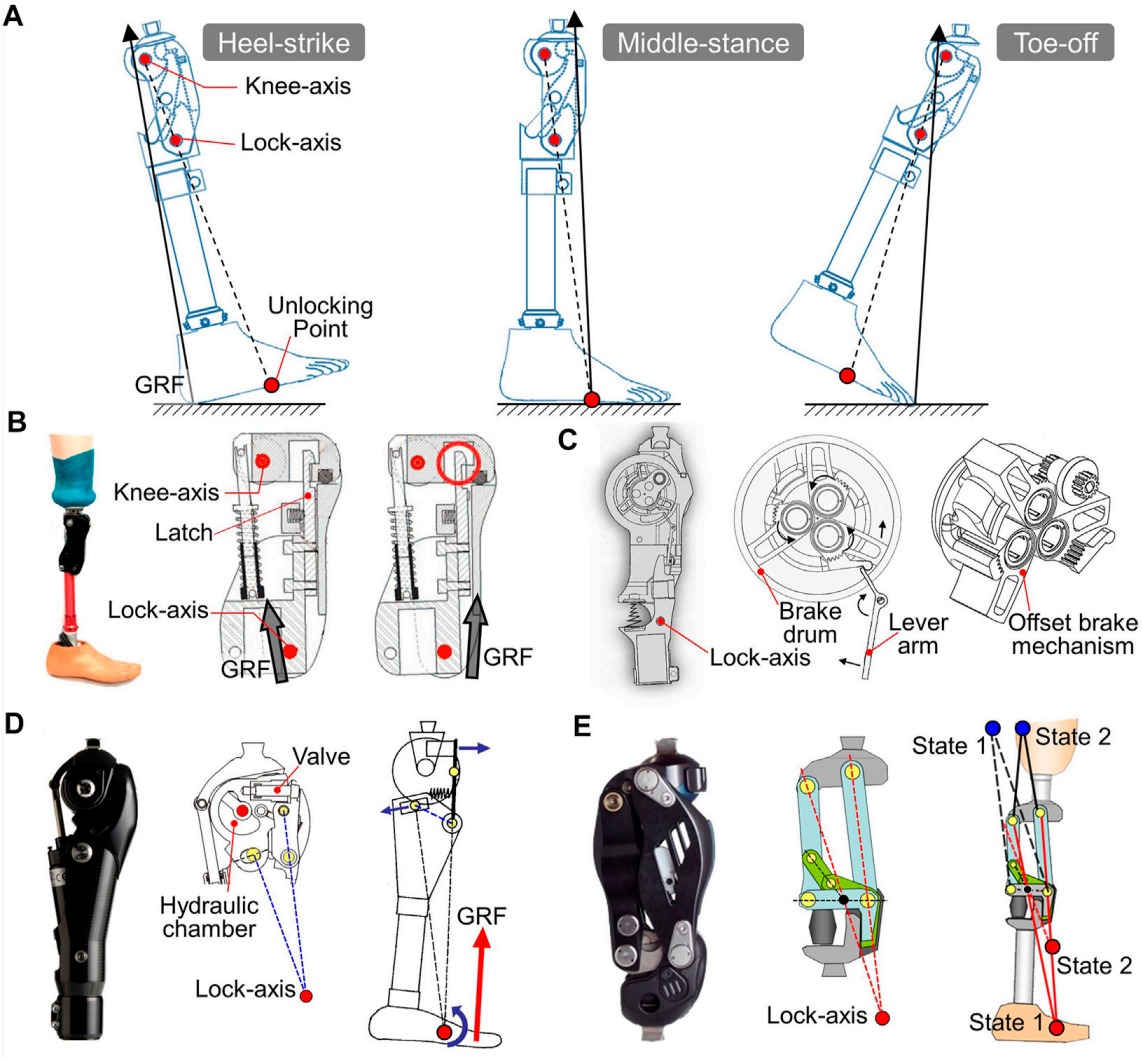
Prosthetic knees with both knee-axis and lock-axis mechanisms for stance stability. **(A)** Effects of lock-axis structure on joint at heel-contact, middle-stance, and toe-off **(B)** Knee with the latch mechanism (SASPL knee, Andrysek et al., 2011), reproduced with permission from copyright 2011 by SAGE. **(C)** Prosthetic knee with the offset brake mechanism (Boiten and Frick, 2017), reproduced with permission from copyright 2019 by Ottobock^®^. **(D)** Knee with the virtual lock-axis based on crank-slider mechanism (Nabtesco^®^ Hybrid, Okuda et al., 2009), reproduced with permission from copyright 2009 by Nabtesco^®^. **(E)** Knee with the virtual lock-axis based on six-bar linkage mechanism (Nabtesco^®^ NK-6 Symphony, Okuda and Nakaya, 2013), reproduced with permission from copyright 2013 by Nabtesco^®^.

The physical lock axis is formed by a pivot to ensure the compactness and controllability of the locking mechanism. The unlocking point is located on the foot and acts as a dividing point for GRF to activate or deactivate the lock mechanism (Figure 8A). Three states occur during the stance phase with the variation in GRF. First, at heel contact, GRF passes posteriorly to the knee axis and lock axis, causing flexion moments at both axes. The lock mechanism is engaged to prevent knee flexion, even with a flexion moment at the knee center. Second, GRF disengages the lock mechanism after the unlocking point caused by middle-foot contact, while the knee axis stays in the extension state due to the extension moment. Third, the GRF passes between the lock axis and knee axis. The lock axis still maintains a disengaging state, while the knee axis rotates because of the flexion moment.

Typically, a latch lock mechanism is used (Figure 8B); it has a strategically positioned lock axis that responds to GRF vectors (Andrysek et al., 2011). The frictional brake can also be used as a lock-axis mechanism. For example, an offset brake mechanism has been used in a prosthetic knee to control a lever arm for releasing the frictional lock (Figure 8C) (Boiten and Frick, 2017).

#### 4.3.3 Virtual lock-axis mechanisms

The physical lock axis needs to move the stump anteriorly during disengagement, which leads to hyperextension movement at midstance and preswing. This results in a small wobble, and the users will feel unstable (Arelekatti et al., 2018b). There is an optimal position of the unlocking point for a specific individual, and the lock axis should be located on the line that connects the knee axis and the optimal unlocking point. The vertical distance between the knee axis and the lock axis is inversely proportional to the hyperextension angle. For the physical lock axis, once its horizontal position is set, the limited vertical distance between the knee axis and lock axis will lead to a large hyperextension angle.

The four-bar linkage mechanism rotates about the ICR, forming a virtual lock axis with a lower position than that of the physical lock axis (Berringer et al., 2017). This gives a greater value of the vertical distance between the knee axis and the lock axis. Similarly, a crank-slider mechanism has been utilized for knees; it regulates a valve for opening or closing the channel of the hydraulic chamber (Figure 8D) (Okuda et al., 2009). The channel is normally closed by a compression spring, and only opens if the GRF vector is anterior to the lock axis.

Although the lower positioned virtual lock axis effectively reduces the hyperextension angle, it also increases the instability zone (Figure 8E). A polycentric knee axis together with a virtual lock axis can resolve the conflicts between the instability zone and hyperextension angle. With a delicately designed six-bar linkage, the knee can flex a few degrees before unlocking (Figure 8E) (Okuda and Nakaya, 2013). The knee axis (blue) and lock axis (red) transfer from state 1 to state 2, which greatly decreases the instability zone. During the unlocking process, the knee axis and lock axis return to positions in state 1, where a great vertical distance still exists to reduce hyperextension angle.

### 4.4 Summary

Stance stability is very important to above-knee users, for whom safety is the main demand (Postema et al., 1997). For manually locking knees, the stability at early stance is guaranteed at the expense of a stiff-legged gait, where no flexion occurs during walking. This greatly decreases the walking speed and increases energy expenditure (Waters et al., 1982; Hanada and Kerrigan, 2001). In general, stance stability is characterized by providing a locking moment after heel strike, which should not impede flexion of the transition from stance to swing (Andrysek et al., 2004). The functional mechanisms of the knee, including the weight-activated brake, the hyperextension-controlled unit, and the polycentric linkage have already eliminated the abnormal stiff-leg gait. Prosthetic knees that integrate braking action and polycentric features can further enhance walking capacity. In fact, the capacity of stance stability mainly depends upon the physical condition of users. The directions of the load line at heel-strike and toe-off stages vary individually with musculature and motor control. The area of the zone of voluntary stability accordingly changes with load lines. A voluntary-controlled polycentric knee is advantageous for active users but may be unstable for less-active users. A functional mechanism independent of the user’s hip moment can achieve a higher level of stability.

Stance stability is dependent on the orientation of the load line with respect to the knee joint axis, which is referred to as the “stability-affecting axis” (Andrysek et al., 2005). Knee-axis-based knees have only one stability-affecting axis, whose stability relies on the “brake” moment strategy or posteriorly aligned knee-axis position. These mechanisms, however, impede knee flexion at late stance, which may lead to stumbling. A new solution is to add one stability-affecting axis to the prosthesis, i.e., GRF affects mechanisms (Andrysek, 2010), that can be precisely positioned with respect to the knee axis and GRF. This feature ensures stability of the knee when it is locked from the early-stance to mid-stance phases, while the unlocking process is executed automatically at preswing due to GRF. A knee with two stability-affecting axes is more stable than that with only one from the perspective of the instability diagram method. However, the optimal position of the lock axis in a prosthetic knee is still not achieved. On the one hand, the characteristics of GRF vary among individuals (Berringer et al., 2017). It is difficult to determine a universally suitable position for knee unlocking. On the other hand, the position of the lock axis is associated with the hyperextension angle and instability zone. These two factors should be considered for a safe gait.

## 5 Mechanisms for early-stance-flexion

There is a prosthetic knee with only a stability mechanism that allows for slight ESF; it utilizes the high resistance of a hydraulic system in the form of a rotary brake (Blumentritt et al., 1998; Lang, 2011) and SNS cylinder (Mauch, 1968). These mechanisms are directly connected to the knee axis; the leak rate of the hydraulic system must be finely tuned. However, this kind of knee still has some problems, such as flexing too slowly or being unable to resist body weight. A requirement that a structure independent of the knee axis should be designed in the prosthetic knee is presented. Therefore, ESF mechanisms are proposed for guaranteeing that a prosthetic knee is able to flex in a limited range without losing knee stability.

### 5.1 ESF axis on monocentric knee

An ESF mechanism independent of the knee axis is proposed for achieving a more stable and natural gait. A knee can flex around the rotation axis of the ESF mechanism (ESF axis), while the knee axis is locked to ensure stability. Furthermore, it does not interfere with the motion of the swing phase.

**FIGURE 9:**
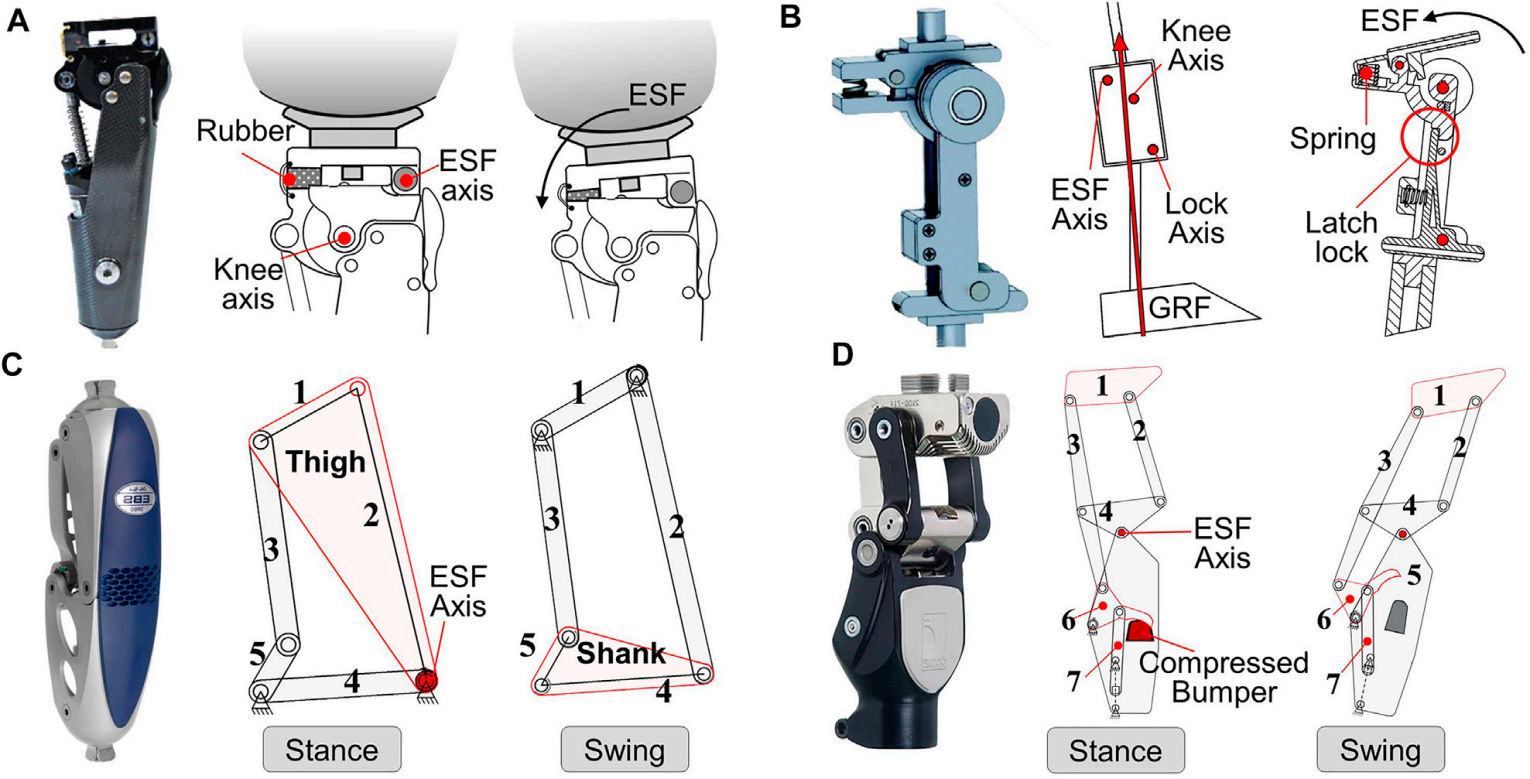
Prosthetic knees with the ESF-axis mechanism. **(A)** Knee with the weight-activated brake and ESF-axis structure (Blatchford^®^ ESK^+^, Blatchford and Tucker, 1980), reproduced with permission from copyright 1980 by Blatchford^®^. **(B)** Prosthetic knee with the lock-axis and ESF-axis structures (Arelekatti and Winter, 2015), reproduced with permission from copyright 2019 by MIT. **(C)** Knee with the ESF-axis mechanism based on five-bar linkage (Ottobock^®^ 3R60, Blumentritt et al., 1997), reproduced with permission from copyright 1997 by Ottobock^®^. **(D)** Knee with the ESF-axis mechanism based on seven-bar linkage (Ossur^®^ Total-2000, Gramnas, 1998), reproduced with permission from copyright 1998 by Ossur^®^.

The ESF axis can work with the knee axis, such as in a weight-activated knee (Figure 9A) (Blatchford and Tucker, 1980). The ESF axis is located anterior to the knee axis and benefits shock absorption and ESF. If GRF moves posterior to the knee axis, the body weight will activate the brake mechanism and block the rotation about the knee axis. Furthermore, rotation about the ESF axis is allowed, where stance flexion is restricted by hard rubber.

The ESF axis can also cooperate with the lock axis, such as in the knee in Figure 9B (Arelekatti and Winter, 2015), where the ESF axis is located posterior to the lock axis. When GRF is posterior to the ESF axis, latching controlled by the lock-axis blocks the knee axis, while the residual thigh flexes relative to the shank about the ESF axis. The flexion of the thigh will be recovered by a spring if GRF translates anteriorly relative to the ESF axis (Arelekatti et al., 2019). This does not affect the releasing process of lock axis at preswing.

### 5.2 ESF axis on polycentric knee

There are also ESF-axis mechanisms in polycentric knees. For example, a redundant five-bar linkage can form two different configurations of polycentric knees in stance and swing. As shown in Figure 9C, link 1 and link 2 are combined to form the thigh during stance, while link 4 and link 5 are combined to form shank during swing (Blumentritt et al., 1997; Grohs et al., 2019). ESF motion with high impedance does not interfere with the swing flexion with low impedance. Similarly, the same functionality is created by a seven-bar linkage mechanism, where the ESF motion is resisted by a bumper (Figure 9D) (Gramnas, 1998).

### 5.3 Summary

At heel-contact, the major function of the knee is impact absorption. During this process, GRF is posterior to the knee axis and causes a large flexion moment. Withstanding a great flexion moment, the thigh extensor muscles must perform negative work, making the knee joint flex at a limited angle within 20° (Murthy Arelekatti and Winter, 2018). The ESF motion allows a person to lower the center of mass of their body during stance, thus absorbing the striking impact force and ensuring the smooth transition from swing to stance. Flexion at early stance is not recommended for the low-function-level (K0∼K2) users, and rotation about the knee axis is accompanied by a high risk of buckling and falling. For knee prostheses without ESF mechanisms, users maintain an extended position during the whole stance phase and utilize the inertia of the trunk to move forward on the support limb. Therefore, it is recommended to use ESF mechanisms in knees of active users (K3–K4 level), which permits ESF in a limited range and simultaneously ensures the stability of the knee axis. The ESF axis controls flexion through resistant elements, including springs, elastic rubber, hard bumpers, and hydraulic absorbers (Arelekatti and Winter, 2015). These passive elements cannot adapt to body weight, speed, or terrain. Unlike a healthy knee, ESF mechanisms can only provide impedance for ESF but no power output for stance extension (Pfeifer et al., 2012). It still cannot replicate the extension torque profile of an anatomic knee due to the absence of adaptivity and energy injection.

## 6 Components for swing resistance

Based on human knee joint biomechanics, the fundamental dynamics of passive damping elements are established. Three kinds of mechanical parts are widely used as swing control elements for knee-axis functional components, including friction, pneumatic, and hydraulic devices.

### 6.1 Frictional components

Friction swing control devices produce damping power for swinging knee via the kinetic friction force between two moving parts. The coefficient of kinetic friction is constant for the same material. This implies that the kinetic friction force can only be adjusted via the applied normal force.

**FIGURE 10:**
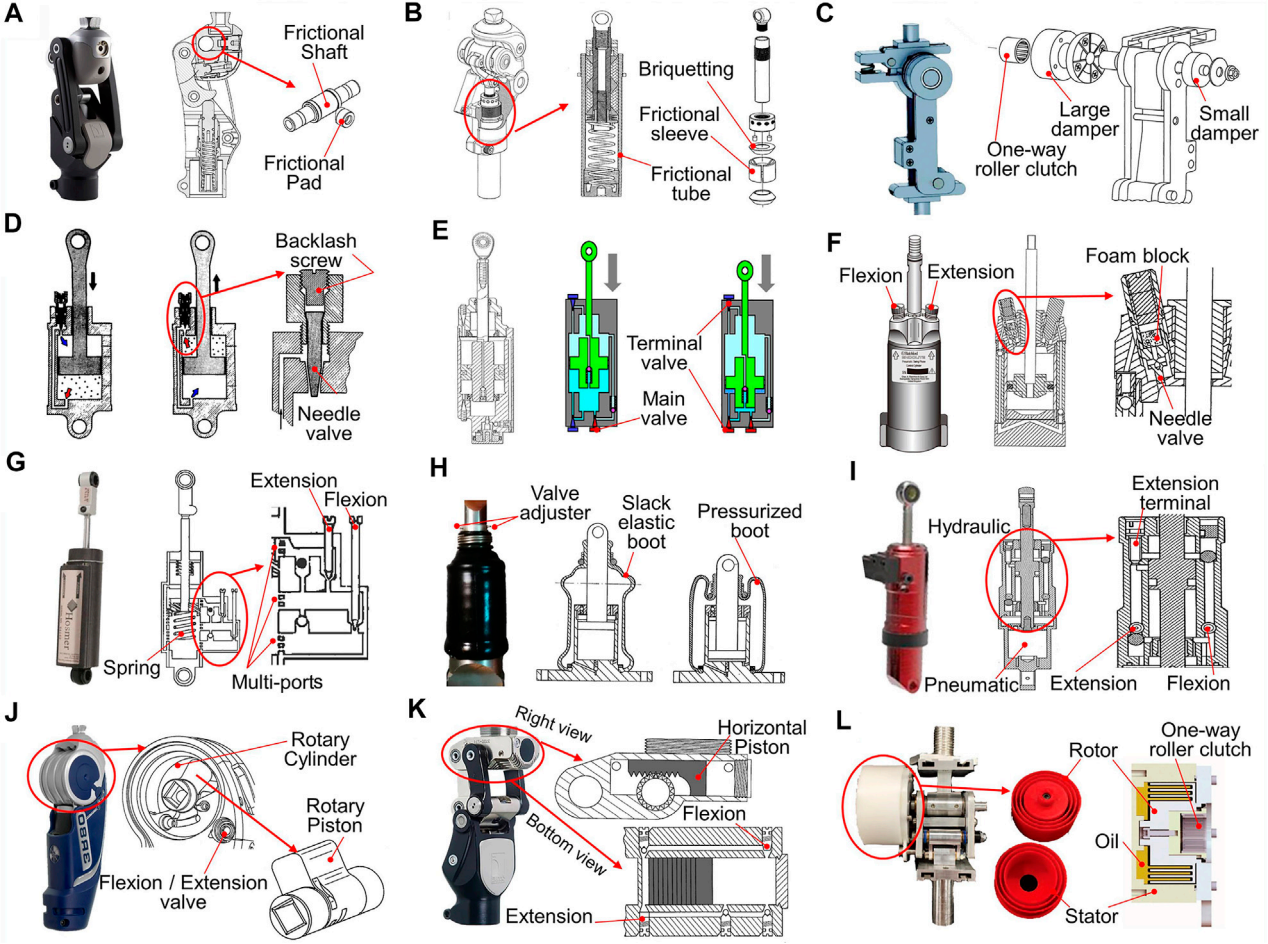
Functional components for swing resistance in passive knees. **(A)** Knee with the frictional elements acting on the pivot axis (Ossur ^®^ Balance, Omarsson et al., 2017), reproduced with permission from copyright 2017 by Ossur^®^. **(B)** Prosthetic knee with the cylindrical frictional elements (Wu, 2011), reproduced with permission from copyright 2017 by **(F)** G. Wu. **(C)** Prosthetic knee with the differential frictional system (Arelekatti and Winter, 2015), reproduced with permission from copyright 2015 by MIT. **(D)** Pneumatic unit with the adjustable backlash at the top of the needle valve (UC-BL knee, Radcliffe and Lamoreux, 1968). **(E)** Pneumatic unit with the terminal phase control valve (Nabtesco^®^ NK-1, Nakaya et al., 2003), reproduced with permission from copyright 2003 by Nabtesco^®^. **(F)** Automatically adjusting pneumatic control unit (Blatchford^®^ ESK^+^ knee, Harris, 1995), reproduced with permission from copyright 1995 by Blatchford^®^. **(G)** Hydraulic cylinder with varying port (Hosmer^®^ Dupaco, Lewis, 1965), reproduced with permission from copyright 1965 by Hosmer^®^. **(H)** Hydraulic cylinder with an elastic boot (Ottobock^®^ Aqua 3WR95, Horvath, 1989), reproduced with permission from copyright 1989 by Ottobock^®^. **(I)** Hydraulic cylinder with air spring (Streifeneder^®^ 3A2500, Krafczyk et al., 2013), reproduced with permission from copyright 2013 by Streifeneder^®^. **(J)** Hydraulic unit with a rotary piston (Ottobock^®^ 3R80, Boiten and Northemann, 2008), reproduced with permission from copyright 2008 by Ottobock^®^. **(K)** Hydraulic unit with a horizontal piston (Ossur^®^ Total-2000, Gramnas, 1998), reproduced with permission from copyright 1998 by Ossur^®^. **(L)** Hydraulic unit that utilizes the viscous friction (MIT GEAR Lab’s knee, Arelekatti et al., 2018a), reproduced with permission from copyright 2018 MIT.

A friction force can be applied on the knee rotation shaft via a friction pad (Figure 10A) (Omarsson et al., 2017), or on the vertical shin tube via friction sleeve and briquettes (Figure 10B) (Wu, 2011). Furthermore, a differential friction-damping system is proposed to fulfill the different moments during swing flexion and extension (Figure 10C) (Arelekatti et al., 2019). It contains two frictional dampers, where the small damper functions during the whole swing phase, and the large damper impedes the knee only during swing extension due to the one-way roller clutch.

### 6.2 Pneumatic components

Pneumatic devices rely on the air compression effect and leak rate and provide a good approximation of the desired knee moment characteristics (Radcliffe, 1977). Typically, pneumatic control devices are based on functional elements of cylinders and pistons. Knee-axis rotation is related to a piston, which leads to a pressure difference between the two sides of the piston that functions as an air spring (Radcliffe and Lamoreux, 1968). The regulated pneumatic differential pressure controls the prosthetic knee motion in a way similar to that of a normal knee. Leak-rate control valves are added to adjust the prosthetic knee moment. The leak rate between the two chambers of the cylinder can change the function of the air spring (Zarrugh and Radcliffe, 1976), which is dependent on the velocity of the piston, the orifice geometry, and thermodynamic properties (Lapera and Yeaple, 1966).

Typically, a pneumatic cylinder is divided into an upper chamber and a lower chamber by a fixed seat, which includes an air-channel orifice area between two chambers that is controlled by an adjustment needle valve (Chen, 1996). The leak rate should also be adjusted in accordance with the requirements of swing flexion and swing extension. A swing control unit with backlash on the top of the needle valve is used (Figure 10D) (Radcliffe and Lamoreux, 1968), where a bigger orifice for air flow can be achieved during extension than during flexion. Furthermore, the impact of terminal flexion and extension can be governed by the terminal valves (Figure 10E) (Nakaya et al., 2003). Pressure-sensitive elements, such as a foam block in the cylinder that can be variably compressed with different pressure intensities (Figure 10F) (Harris, 1995), can cooperate with different walking speeds. Therefore, the resistance of the pneumatic cylinder is adaptive to the user’s walking speeds.

### 6.3 Hydraulic components

The oil in the hydraulic control cylinder is regarded as an incompressible fluid; hence, it can produce a larger force than the pneumatic cylinder. The damping force of a hydraulic device results from the restricted oil that passes the throttle ports in the closed loop. The differential pressure is determined by the flow rate. The damping force can be adjusted by varying the flow area of the throttle port. The damping force exerted by the hydraulic cylinder is linear to the square of the piston velocity, which means that the hydraulic cylinder can also respond to the walking speed voluntarily.

To reduce the user’s burden, a low damping force is required at the initial stage of the flexion/extension phase, while a greater resistant force is essential at the end stage for limiting heel rise or absorbing the impact. The damping force can be adjusted by varying the flow area of the throttle port. For example, the multiple ports in a cylinder change automatically with the position of the piston (Figure 10G) (Lewis, 1965). In the cylinder, a mechanical spring located at the bottom is usually used for motion recovery, while the configuration of the elastic boot surrounding a spring can simultaneously recover the piston and decrease the size of the cylinder (Figure 10H) (Horvath, 1989). Moreover, a pneumatic spring is proposed to replace the mechanical spring in the hydraulic device, where the pneumatic extension-assist system achieves a compliant and comfortable damping effect without the design of multiple ports (Figure 10I) (Krafczyk et al., 2013).

Rotary hydraulic units are adapted to a prosthetic knee within a limited space, which can satisfy the swing of a knee with high damping torque characteristics. In a monocentric knee, the rotary piston flexes with the knee-axis shaft, and the valve inside the channel can regulate the torque profile (Figure 10J) (Boiten and Northemann, 2008). Similarly, in a polycentric knee, one of the pivot shafts is connected to a horizontal piston in the form of a gear rack structure, where the rotation of knee transforms into a horizontal movement of the piston (Figure 10K) (Gramnas, 1998). In addition, viscous friction forces can be generated via the relative motions of the circular fins of the rotor and stator, where silicon oil is filled in annular gaps (Figure 10L) (Arelekatti et al., 2018a).

### 6.4 Summary

The knee is a net power dissipater during the swing phase of level walking. The passive damper elements can replicate the knee power during swings. With a proper valve design and the specific position of the orifice in the fluid circuit, pneumatic and hydraulic dampers can achieve angle-torque profiles that are very similar to those of a physiological knee. The damping effects govern the heel rise of swing flexion and absorb the impact at the end of swing extension, which ensures a smooth transition from swing to stance. However, these passive devices cannot provide natural ambulation on diverse walking surfaces, such as ramps and stairs (Chin et al., 2006). There was no significant difference in energy consumption between the passive knee and MPK at a self-selected walking cadence (Schmalz et al., 2002; Johansson et al., 2005).

## 7 Discussion

In this review, representative passive knee mechanisms are reviewed according to biomechanical requirements. Furthermore, we designed Table 2, which includes current commercial and recently studied passive knees, for users or developers to understand and analyze the functional mechanisms and components from the perspective of stability, ESF, and swing resistance. Accordingly, we present ideas about three general trends in the current and future development of prosthetic knees.

**TABLE 2:** Passive prosthetic knees based on walking functions.

Functional mechanisms and components of passive knees				Swing resistance					
				Frictional	Pneumatic		Hydraulic		
							Rotary	Linear	
Stance	Stance Stability	Monocentric Knees	Manual-Lock					OT-3R95, OT-3WR95	
			Weight-Brake	OT-3R49	OT-3R92, OS-OP4, NA-NK1				
			Hyperextension-Controlled					OS-Mauch, BL-Mercury	
		Polycentric Knees	Elevated or	OT-3R30, OS-Balance	OT-3R106, OT-3R78, OS-Paso, OS-OHP3, BL-S500, ST-3A1800		OS-Cheetah	OT-3R67, OT-3R55, OS-OH5, OS-OH7, TL-X6, ST-3A2500	
			Hyper-stabilized or										
			Voluntary Controlled										
			Four-Bar Linkage + Weight-Brake		DAW-Sure Stance,				
			Four-Bar Linkage + Hyperextension-Controlled					BL-KX06	
		Knees + Lock	SA (Knee) + SA (Lock)	LCKnee (Andrysek et al., 2011)					
			SA (Knee) + 4-Bar Linkage (Lock)		NA-Hybrid				
			Linkage Mechanisms					NA-NK6	
	Stance, Stability and ESF	Knees + ESF	SA (Knee) + SA (ESF)		BL-ESK				
			Linkage Mechanisms	OT-3R62, OS-Total1900	TL-5PSOH		OS-Total2000	OT-3R60	
		Knees + Lock + ESF	SA (Knee) + SA (Lock) +SA (ESF)	MIT-Knee-1 (Murthy Arelekatti and Winter, 2018)			OT-3R80		
			SA (Knee) + Linkage mechanisms (Lock) +SA (ESF)				MIT-Knee-2 (Berringer et al., 2017)		

NOTE: SA, single axis; OT, Ottobock®; OS, Ossur®; BL, Blatchford®; NA, Nabtesco®; TL, Tehlin®; ST, Streifeneder®.

### 7.1 Adaptivity

Passive knee research mainly concentrates on the biomechanics of level walking. However, passive knees cannot meet the needs of the users’ daily activities. The adaptivity of knee prostheses should be improved from two aspects.

#### 7.1.1 Adaptivity to the environment.

Prosthetic knees are expected to deal with environmental elements including irregular terrain, ramps and stairs. Microcontrollers have been introduced to MPKs and allow automatic variation in the damping, which can accommodate a wider range of environmental factors. However, most MPK solutions are monocentric and are typically based on a single knee-axis structure. The knee-axis-based hydraulic unit of the MPK is required to provide adequate damping for stance flexion and stance stability, simultaneously. Thus, compared to a healthy knee, asymmetric gait with a smaller stance-flexion angle arises in MPKs (Thiele et al., 2019). The adaptivity can be improved by combining microprocessor-controlled units and passive mechanisms; for example, stance stability and ESF can be controlled by automatically adjusting the structures of knee axis and ESF mechanism, and the swing resistance can be regulated by microprocessor-controlled units. The functional components acting on different phases can be automatically adjusted to the optimized state according to the environmental factors without interfering with each other. In addition to the MPK solutions, the adaptivity can be enhanced only by passive mechanisms. A passive mechanism that acts as a lock axis has been added to a knee device; it locks the knee and generates an extension moment around the knee axis during the stance phase without using any actuators (Inoue et al., 2013). This mechanism enables the knee to adapt to stair ascent, which is based on the knowledge that GRF translates and increases when stance flexion occurs. Other mechanisms or intelligent units may be integrated with current passive knee, which can be further developed and optimized.

### 7.1.2 Adaptivity to users

Passive knees are not capable of recognizing an individual’s intent and can only use pneumatic and hydraulic units to change the damping force in a limited range with changing walking speeds. The estimation or recognition of a user’s locomotive intent is more important in state-of-the-art prosthetic knees, which can directly adapt for different speeds, terrain, and obstacles. Biomechanical instrumentation comprising angles, loads, and inertial sensors is commonly used in MPKs and APKs, which collect kinematics and force signals to match the predefined locomotive states. These signals are stable and highly repetitive, which makes the finite state machine (FSM) control strategy capable of commanding the knee to a robust and well-defined state. However, there is hysteresis in the FSM strategy (Martin et al., 2010). The locomotive state knowledge with sensor-based information comes from previous steps, and the angle and damping of the joint may not be best suited for the immediate current step. Furthermore, the sensor signals only reflect the movement of the prosthesis, not the intentions of users. It is still a limited framework that cannot adapt to arbitrary motions of the user. Non-invasive electromyography (EMG) is another method that is used as volitional control, but the weak signal amplitude, noise during acquisition, and muscle deficiency of the residual limb all restrict the quality and robustness of EMG. Thus, it appears to be less appropriate and far from being a stand-alone technology for dynamic locomotion. On the other hand, the EMG-based approach combining the embedded sensors exhibits higher adaptivity and stability (Peeraer et al., 1990; Au et al., 2008). In the authors’ opinion, functional mechanisms and components are closely associated with walking biomechanics, and variation in locomotive states can be straightforwardly mapped to the functional axis in real time. For instance, a mechanical sensor mounted on a lock-axis structure can perceive the transition from stance to swing immediately. Feedforward or feedback can be achieved by adjusting the position of the virtual lock axis. The mechanical intelligence used for the adaptive prosthesis–user interaction remains a possibility in the future.

### 7.2 Controlled energy flow

Daily activities, such as running, jumping, or stair climbing, require significant amounts of energy input, thus leading to the need for APKs (Jacobs et al., 1996; Riener et al., 2002). Some of the latest prototypes have already improved kinematics for normal gait, which have even approached biological levels (Lawson et al., 2014; Zhao et al., 2017). However, an active prosthesis is normally heavier than a passive prosthesis, which leads to the primary drawback of a higher metabolic cost for the users (Pfeifer et al., 2015).

Passive knees are lightweight and energy-efficient because the mechanisms and components are highly matched to walking biomechanics. Therefore, one of the challenges in the future is how lightweight and effective functional mechanisms can be integrated into actuators to minimize user metabolic costs. Some novel actuator designs have already demonstrated progress in achieving this objective and have high-efficiency and elastic-compliant actuators that reduce the overall weight of the prosthesis (Pieringer et al., 2017). In these knee designs, there are similar principles between elastic actuators and passive mechanisms. For instance, the weight acceptance (WA) actuator in the CYBERLEGS Beta-Prosthesis knee provides the same functions as the ESF-axis mechanism in the passive knee (Flynn et al., 2018). The WA system locks a high-stiffness spring via a nonbackdrivable screw during loading, allowing stance flexion, while it can be disengaged by a low-powered motor without interfering with swing locomotion of the knee. In addition, the electromagnetic clutch in the CESA knee can be engaged or disengaged for blocking or enabling the swing flexion of the knee and acts quite the same as the lock-axis mechanism (Rouse et al., 2013). Integrating energy-storage mechanisms into actuators can be a promising design solution, since they help to develop small but powerful prostheses that can offer more natural gait due to compliant behavior and decreased weight. Because the “negative” work at the knee is greater than the “positive” work, a whole energy regenerative solution is still a challenge (Laschowski et al., 2019). The mismatch between the input and output energies in current knee devices indicates the difficulty of achieving high efficiency in a simple mechanism. This confirms that as the magnitude of the positive energy demand increases, the supplementary mechanisms that control energy-storage elements become more important.

### 7.3 Knee design specification

Prosthetic knee specification is lacking, with only one international standard (ISO10328) available for structural fatigue testing (Lara-Barrios et al., 2018). Various structures and components with different functions have increased the complexity of knee prostheses. It also increases the difficulty for users, doctors, and prosthetists to find updated knowledge on the latest developed prosthetic knee technologies. Thus, it is difficult to understand the relationships between knee functions and mechanisms, resulting in barriers to appropriate adjustment and ideal states. In addition, according to the author’s experience, a knee prosthesis is a vulnerable product after 3–5 years of use. If one of the functional structures or components breaks down, the entire knee prosthesis is discarded. The level of maintenance and interchangeability of knee prostheses is far from that of in industrial parts and products. This greatly increases the economic burden on users, and it is essential to improve the service life of knee prostheses.

In this review, we proposed the concept of functional mechanisms and components, not only to determine the explicit relationship between knee functions and structures of prostheses, but also to promote the construction of specifications and standards for prosthetic knee design. We suggest that the design of functional mechanisms and components be tailored to the lost functions of users. The components acting on the same functional axis are supposed to be interchangeable and easily installed, even if these parts may be made by different manufacturers.

Furthermore, the concept of functional mechanisms and components is intended to facilitate the development of knee prostheses. Typically, an intelligent knee prosthesis requires the integration of multidisciplinary knowledge, including human neuroscience, biomechanics, mechanical design, electronic design, motion control, and signal processing. To remove the barrier and facilitate progress in knee prosthesis research, a commonly used platform is desired. Thanks to open-source models, such as the open-source leg developed by the University of Michigan, researchers can directly test their control algorithms (Azocar et al., 2020). From the perspective of widely used products, designing a prosthetic knee should start from the basic functions, and the knees should be designed with lightweight and compact functional mechanisms. We aim to construct a framework that provides a theoretical system for those who are less aware of the structures and biomechanics of prosthetic limbs, thus accelerating the development and clinical testing of prosthetic knees.

## 8 Conclusion

This review provides a new paradigm of prosthetic knee analysis, which clearly outlines the complex mechanisms of diverse knee prostheses and builds straightforward relationships between prosthetic knee structures and human walking biomechanics. First, the main function of prosthetic knees is to maintain stability during the stance phase. The monocentric mechanisms, polycentric mechanisms, and GRF-affected mechanisms in passive knees are introduced. These mechanisms can satisfy the requirement of stance stability and avoid buckling at an early stance or stumbling at a late stance. Second, ESF is desired for shock absorption and leg braking in active (K3–K4) users. There are ESF mechanisms in passive knees that allow a limited flexion angle at the heel-strike stage without losing stability. Third, knee prostheses need to regulate the maximum flexion angle and eliminate end impact during the swing phase, thus achieving an energy-saving natural gait. The frictional, pneumatic, and hydraulic components that control the motion during the swing phase are listed.

The passive mechanisms and components provide a new perspective based on the biomechanical functions, and the mechanical structures of passive knees can be used and controlled independently without interfering with each other. This new insight enables the interchangeability of prosthetic knee structures and components. By replacing an unsuitable part, the performance of the whole knee prosthesis can be improved. Furthermore, it is possible to consider the connections between passive mechanisms and walking biomechanics in the design of semiactive and active knee prostheses. The actuation, sensing, and control units can be simplified by mechanical parts that intrinsically match human knee biomechanics. The hardware of an intelligent prosthetic knee is supposed to be achieved by integrating the functional mechanical parts, low-powered actuation system, and precise sensor elements.

## Nomenclature

***M***_***he***_: extension moment of the hip joint
***F***_***sb***_: shear force at the center of pressure (COP) of foot during heel contact
***L***_***b***_: total length of the residual leg from hip joint to the prosthetic heel
***M***_***bk***_: braking moment exerted by prosthetic knee
***F***_***lb***_: load force carried along the hip-COP line
***F***_***rb***_: load force resulted from $F_{lb}$ and $F_{sb}$
***X***_***b***_: vertical distance from the knee joint to hip-COP line
***Y***_***b***_: vertical height of the knee instantaneous center rotation at heel-contact
***M***_***hf***_: flexion moment of hip joint
***F***_***ls***_: load force carried along the hip-COP line at leg stumble
***F***_***ss***_: shear force at the COP at toe-off
***F***_***rs***_: load force resulted from $F_{ls}$ and $F_{ss}$
***L***_***s***_: total length of the residual leg from hip joint to the prosthetic toe
***X***_***s***_: vertical distance from hip-COP line to knee joint (inverse to $X_{b}$ in direction)
***Y***_***s***_: vertical height of knee instantaneous center rotation at toe-off
